# Etiology-based scoring for pediatric secondary intussusception: a retrospective analysis of clinical heterogeneity

**DOI:** 10.3389/fped.2025.1624050

**Published:** 2026-01-15

**Authors:** Jinfang Sun, Haiyan Hu, Sanli Fan, Yan Qin, Xiaoling Meng

**Affiliations:** Department of Pediatric Gastroenterology, Shanxi Children’s Hospital, Taiyuan, Shanxi, China

**Keywords:** artificial intelligence, diagnostic model, pathological lead point, pediatric intussusception, retrospective study, scoring system

## Abstract

**Purpose:**

To investigate clinical heterogeneity in pediatric secondary intussusception and to develop four simplified, etiology-specific scoring systems to facilitate preoperative etiologic prioritization after intussusception has been confirmed.

**Methods:**

This retrospective study analyzed 92 pediatric patients diagnosed with secondary intussusception from 2018 to 2023. Disease patterns across PLP subtypes were analyzed using a four-dimensional framework including etiology, age, sex, and clinical features. Candidate variables were selected based on clinical plausibility and univariable screening before being entered into etiology-specific OvR models. Etiology-specific scoring models for IgA vasculitis (IgAV), Meckel's diverticulum (MD), intestinal polyps (IP), and intestinal duplication (ID) were constructed using binary logistic regression and a one-vs.-rest strategy. Each score was internally validated according to ROC curves, with discrimination assessed by AUC as well as sensitivity and specificity.

**Results:**

The IgAV score incorporated hematochezia (+2), age ≥6 years (+1), and absence of abdominal mass (+1), with an AUC of 0.85, sensitivity of 80%, and specificity of 75.4%. The AUCs for Meckel's diverticulum, intestinal polyps, and intestinal duplication were 0.780, 0.925, and 0.851, respectively. The remaining etiology-specific scores similarly comprised two to three bedside-available variables (e.g., demographic features and key clinical manifestations), allowing for practical use in the preoperative setting.

**Conclusion:**

These simple, non-invasive scoring systems may assist early etiologic prioritization and support more targeted diagnostic assessment in pediatric intussusception. Their simplicity and internal performance characteristics suggest potential utility in acute care settings. Future studies are warranted to validate their generalizability and to explore integration into routine clinical workflows.

## 1 Introduction

Secondary intussusception, defined by the presence of a pathological lead point (PLP), represents a clinically important but diagnostically challenging abdominal emergency in children, accounting for approximately 5%–10% of all intussusception cases (1, 2). Delayed or inaccurate identification of the underlying PLP may result in bowel ischemia, necrosis, perforation, or peritonitis, thereby substantially increasing surgical morbidity and mortality (2–5). Common PLP etiologies in pediatric populations include Meckel's diverticulum (MD), IgA vasculitis (IgAV), intestinal polyps, intestinal duplication, and less frequently neoplastic lesions (1, 6–8, 10–12). Importantly, these etiologies differ not only in pathophysiology but also in age distribution, clinical presentation, and disease course, giving rise to marked heterogeneity in presentation and management.

Despite increasing recognition of etiologic diversity in secondary intussusception, most existing studies have analyzed PLP-related cases as a single clinical entity or in a binary manner (PLP present vs. absent), rather than conducting systematic comparisons across specific PLP subtypes. Large retrospective analyses have predominantly focused on downstream outcomes such as recurrence (9), bowel resection (2, 5, 10, 11), or failure of nonoperative reduction (12, 13), without stratifying patients by distinct etiologies. As a result, clinically relevant differences in age distribution, sex predominance, symptom profiles, and associated features among MD-, IgAV-, polyp-, or duplication-related intussusception remain incompletely characterized. Moreover, current predictive and scoring models are largely outcome-oriented rather than etiology-driven, limiting their utility in supporting early bedside etiologic prioritization once intussusception has been established.

To address these gaps, we conducted a retrospective analysis of pediatric secondary intussusception using a multidimensional “etiology–age–sex–symptom” framework to systematically characterize clinical heterogeneity across major PLP subtypes. Age and sex were incorporated as developmental and epidemiologic priors, whereas symptom patterns were selected to reflect bedside-available phenotypes assessable after confirmation of intussusception, a common clinical scenario in which imaging establishes intussusception but provides variable ability to characterize the PLP. Based on this framework, we further developed four simplified, etiology-specific scoring systems to support early preoperative etiologic prioritization and diagnostically informed decision-making. By aligning structured heterogeneity analysis with pragmatic, bedside-oriented tools, this study aims to provide a more individualized and clinically actionable strategy for the evaluation of pediatric secondary intussusception.

## 2 Methods

### 2.1 Study population

This retrospective cohort study enrolled pediatric patients diagnosed with secondary intussusception at Shanxi Children's Hospital between October 2018 and October 2023. Ethical approval was granted by the hospital's Institutional Review Board. Inclusion criteria mandated: (1) a confirmed diagnosis of intussusception established via abdominal ultrasound, air enema reduction, surgical exploration, or endoscopic examination; (2) definitive evidence (pathological or imaging-based) of an organic pathological lead point (PLP); and (3) availability of comprehensive medical records detailing clinical symptomatology, treatment course, and follow-up data. Patients were excluded if they presented with: (1) primary (idiopathic) intussusception; (2) significant comorbid congenital intestinal anomalies (e.g., Hirschsprung's disease) or major systemic conditions (e.g., congenital heart disease, immunodeficiency); or (3) critical data omissions, such as unverified etiology or unrecorded treatment outcomes.

Following these criteria, 92 children were ultimately enrolled. Based on the identified PLP, participants were categorized into five etiological groups: Meckel's diverticulum (MD, *n* = 27), intestinal duplication (ID, *n* = 12), IgA vasculitis (IgAV, *n* = 25), intestinal polyps (IP, *n* = 13), and an “Others” group (*n* = 15). This “Others” group was inherently heterogeneous, comprising the following specific diagnoses: intestinal lymphoma (*n* = 6), enterogenous cyst (*n* = 3), inflammatory mass in the ileocecal region (*n* = 2), allergic vasculitis (*n* = 1), abdominal urticaria (*n* = 2) and gastric volvulus (*n* = 1).

### 2.2 Variable definition and classification

The study assessed multiple variables as follows: (1) Etiological classification of PLPs, including Meckel's diverticulum, intestinal duplication, IgA vasculitis, intestinal polyps, and other causes. (2) Gender was documented as a dichotomous parameter (male or female). (3) Age was treated as both a continuous and categorical variable, stratified into four developmental phases: infancy (<1 year), early childhood (1–<3 years), preschool (3–<6 years), and school-aged children (≥6 years). (4) Clinical manifestations—such as vomiting, abdominal discomfort, blood in stool, fever, detectable abdominal mass, and electrolyte imbalances—were all recorded as binary variables indicating presence or absence. Skin changes were specifically defined as the presence of purpuric rash, urticaria, or mucocutaneous pigment spots (oral, perianal, extremities). Joint involvement was defined as swelling or pain localized to the extremity joints. (5) Symptom duration was defined as the time interval (in hours) from caregiver-reported symptom onset to diagnostic confirmation of intussusception.

### 2.3 Etiological diagnosis: standards and procedures

Etiological diagnoses were established through a synthesis of clinical presentation, imaging findings (including ultrasound, contrast studies, and computed tomography), intraoperative findings, and histopathological confirmation. For 29 patients, the underlying primary condition had been diagnosed prior to the intussusception episode based on characteristic clinical features, including 25 cases of IgA vasculitis (IgAV) presenting with typical purpuric rash with or without arthralgia and abdominal symptoms, 2 cases of abdominal urticaria, 1 case of allergic vasculitis, and 1 case of gastric volvulus confirmed by upper gastrointestinal contrast study.

In 56 children, the PLP etiology was definitively identified via surgical exploration and subsequent pathological examination of resected specimens (confirming MD, ID, enterogenous cysts, Peutz-Jeghers polyps, familial adenomatous polyposis-related polyps, inflammatory masses, solitary polyps, or lymphoma). For the remaining 7 patients, diagnosis was achieved through colonoscopy with biopsy and histopathology.

To ensure clear etiological classification and avoid conceptual overlap, IgAV-associated intussusception was defined strictly according to established diagnostic criteria (EULAR/PRINTO/PRES), requiring the presence of characteristic purpuric rash with or without arthralgia, abdominal involvement, and/or renal manifestations. In these cases, bowel wall edema secondary to IgA-mediated small-vessel vasculitis was considered a functional pathological lead point, and alternative structural PLPs were excluded through appropriate imaging and/or surgical or endoscopic evaluation.

In contrast, cases labeled as abdominal urticaria or allergic vasculitis did not meet the diagnostic criteria for IgAV and lacked evidence of IgA-mediated systemic vasculitis. These conditions were diagnosed based on clinical presentation and supportive imaging findings consistent with localized allergic or inflammatory processes. Given their distinct pathophysiological mechanisms, limited case numbers, and marked clinical heterogeneity, these entities were classified within the “Others” group rather than combined with IgAV.

Similarly, gastric volvulus–associated intussusception was included as secondary only when the diagnosis was confirmed by characteristic imaging findings and no concurrent structural PLP was identified. This multi-pronged diagnostic and exclusion strategy ensured a high level of accuracy and internal consistency in etiological classification across all study groups.

### 2.4 Statistical analysis

A retrospective cross-sectional analysis was conducted to evaluate the demographic characteristics, etiology distribution, and clinical features among pediatric patients diagnosed with secondary intussusception at our institution. Patient demographics and clinical variables were stratified according to specific pathological lead points (PLPs). Between-group comparisons across major PLP categories, including age, sex, and key clinical manifestations, were predefined as the primary analyses to characterize etiologic heterogeneity, whereas within-group assessments of age- and sex-related modifiers (Section 3.3) and distributional shape testing of age (e.g., dip test) were conducted as secondary exploratory analyses.

Categorical variables, including gender, age group, and presence or absence of clinical symptoms, were analyzed using either Chi-square or Fisher's exact tests based on sample size and data characteristics, to determine differences among various PLP categories or between demographic subgroups. Continuous variables, such as patient age, were compared using the Kruskal–Wallis test. *Post-hoc* pairwise comparisons using the Mann–Whitney U test were conducted following significant findings, with corrections applied for multiple comparisons where indicated [Bonferroni or False Discovery Rate (FDR)].

Non-parametric unimodality testing was performed to explore the shape of age distributions within PLP subgroups. Specifically, Hartigan's dip test was applied to the age distribution of the Meckel's diverticulum subgroup to assess evidence of multimodality (e.g., a bimodal pattern), guided by visual inspection of kernel density plots. The dip test was implemented in R (version 4.4.2) using the diptest package. Given its exploratory nature, dip-test results were interpreted descriptively and were not used for variable selection or model development.

Statistical significance was set at a two-tailed *P*-value of less than 0.05. Statistical processing and analysis were performed utilizing SPSS software (IBM, version 21.0) and R statistical software (version 4.4.2).

### 2.5 Development and validation of scoring systems

To facilitate preoperative differentiation among major pathological lead points (PLPs), we developed four simplified, etiology-specific scoring systems targeting IgA vasculitis (IgAV), Meckel's diverticulum (MD), intestinal polyps (IP), and intestinal duplication (ID). Guided by the etiology–age–sex–symptom framework, candidate predictors were restricted to routinely obtainable bedside variables at initial assessment (age/sex and presenting symptoms/signs), with the aim of supporting etiologically oriented triage and diagnostic prioritization rather than outcome-only prediction.

Candidate variables were initially screened using univariate analyses appropriate to data type and distribution. Variables demonstrating statistical significance or strong clinical plausibility based on prior literature were entered into etiology-specific one-vs.-rest (OvR) logistic regression models, in which the target PLP was coded as the outcome of interest (“1”) vs. all other etiologies (“0”). For subgroups with limited sample size (e.g., IP and ID), Firth's penalized likelihood estimation was applied to reduce small-sample bias and separation.

Regression coefficients (*β*-values) from the final models were standardized and proportionally scaled, and integer point values were assigned by rounding to the nearest whole number to preserve the relative contribution of predictors while ensuring bedside usability. Specifically, coefficients were divided by the smallest absolute non-zero *β* within each final model and multiplied by a constant to yield small integers; scaled coefficients were then rounded to the nearest integer, with negative coefficients retained as negative point values where applicable (e.g., IP and ID scores).

Model performance was evaluated by plotting receiver operating characteristic (ROC) analysis with the area under the curve (AUC) as the primary metric. Optimal cut-off scores for each system were determined using the maximum Youden index (sensitivity + specificity − 1). Internal validation was performed using bootstrap resampling with 1,000 iterations to estimate optimism and assess the risk of overfitting; for each bootstrap sample, the model was refit and evaluated both in the bootstrap sample and in the original dataset, and the mean difference in AUC was taken as the optimism bias estimate to assess the risk of overfitting. Performance metrics reported in this study represent apparent discrimination in the derivation cohort. External validation using an independent cohort was not performed and remains a priority for future multicenter prospective studies.

### 2.6 Clinical interpretation of predictors

Each predictor included in the final scoring systems was selected not only for statistical significance but also for clinical plausibility. For instance, the absence of a palpable abdominal mass was frequently observed in IgAV-related intussusception, likely due to diffuse mucosal edema rather than localized lesions. In contrast, the biphasic age distribution seen in Meckel's diverticulum reflected both embryological origins and symptomatic timing, justifying its inclusion in the MD-specific model.

### 2.7 Application of the scoring systems

Diagnostic performance was evaluated by plotting ROC curves and calculating AUCs. Scores achieved AUCs ranging from 0.780 (MD) to 0.925 (IP), demonstrating satisfactory to excellent discrimination. Youden's index was used to determine optimal cutoff values, and corresponding sensitivity and specificity were reported. A clinical application flowchart (Figure 3) was constructed to guide practical implementation: for example, an IgAV score ≥3 indicates the need for early immunologic workup, while an IP score ≥0 suggests prompt colonoscopic evaluation.

## 3 Results

### 3.1 Cohort characteristics

Among the 92 enrolled pediatric patients with confirmed secondary intussusception, males were predominant (63.0%), school-aged children (≥6 years) accounted for the largest age subgroup (42.4%), and Meckel's diverticulum (29.3%) and IgA vasculitis (27.2%) were the most common pathological lead points (PLPs); abdominal pain (83.7%) and vomiting (63.0%) were the most frequent presenting symptoms, and all diagnoses were confirmed by surgery, endoscopic evaluation with histopathology, and/or radiologic assessment, in conjunction with established clinical diagnostic criteria where applicable.

The detailed demographic and clinical characteristics of the cohort are summarized in Table 1. The median age was 3.5 years (range: 2 months–14 years), with a distribution skewed toward older children, and school-aged patients (≥6 years) constituted the largest age subgroup. Regarding etiological composition, Meckel's diverticulum and IgA vasculitis were the two most common pathological lead points. Additional clinical manifestations included a palpable abdominal mass (53.3%), hematochezia (48.9%), fever (29.3%), anemia (18.5%), and electrolyte imbalance (20.7%).

**Table 1:** Clinical features and variables in pediatric secondary intussusception.

Variables	Types	Frequency	Proportion (%)
Sex	Male	58	63
	Female	34	37
Age Groups	Infancy (0–1 years)	12	13
	Toddler (1–3 years)	17	18.5
	Pre-school (3–6 years)	24	26.1
	School-age and above (≥6 years)	39	42.4
PLPs	Meckel's diverticulum	27	29.3
	IgA vasculitis	25	27.2
	intestinal polyps	13	14.1
	intestinal duplication	12	13.0
	Others	15	16.3
Main Clinical Manifestations (Present)	Vomiting	58	63
	Abdominal Pain	77	83.7
	Hematochezia (Blood in stool)	45	48.9
	Palpable Mass	49	53.3
	Fever	27	29.3
	Anemia	17	18.5
	Electrolyte Imbalance	19	20.7
	Arthralgia	10	10.9
	Skin Changes	26	28.2

### 3.2 Etiology-specific clinical features

Marked variations were identified among different PLP categories in terms of patient age (*p* = 0.009), duration of symptoms before diagnosis (*P* = 0.009), anatomical subtype of intussusception (*p* < 0.001), as well as the incidence of clinical symptoms, notably abdominal pain (*p* < 0.001), emesis (*P* = 0.036), and electrolyte abnormalities (*P* = 0.028) (Table 2). Specific distinguishing clinical characteristics for each PLP category are summarized in Table 3.

**Table 2:** Comparison of clinical characteristics among PLPs groups.

Variable	Data type	Statistical method	*P*-value
Age	Continuous	Kruskal–Wallis Test	**0**.**009**
Sex	Categorical	Chi-square Test	0.117
Duration of symptoms			**0**.**009**
Type of intussusception			**<0**.**001**
Vomiting			**0**.**036**
Abdominal pain			**<0**.**001**
Hematochezia			0.08
Palpable abdominal mass			0.083
Fever			0.126
Anemia			0.908
Electrolyte imbalance			**0**.**028**

Bold values indicate statistical significance (*P* < 0.05).

**Table 3:** Distribution of Key clinical features by PLPs category and Age group.

PLPs	Age Ggoup (years)	Total (*N*)	Sex (Female/Male, *n*)	Vomiting [*n* (%)]	Abdominal pain [*n* (%)]	Electrolyte imbalance [*n* (%)]
MD	0–1	3	1/2	2 (66.7)	2 (66.7)	2 (66.7)
	1–3	8	2/6	6 (75.0)	7 (87.5)	4 (50.0)
	3–6	7	3/4	6 (85.7)	7 (100.0)	2 (28.6)
	≥6	9	1/8	6 (66.7)	8 (88.9)	1 (11.1)
Intestinal polyps	0–1	3	3/0	3 (100.0)	0 (0.0)	0 (0.0)
	1–3	2	0/2	0 (0.0)	0 (0.0)	0 (0.0)
	3–6	3	0/3	0 (0.0)	1 (33.3)	0 (0.0)
	≥6	5	1/4	3 (60.0)	4 (80.0)	0 (0.0)
Intestinal duplication	0–1	2	2/0	1 (50.0)	2 (100.0)	1 (50.0)
	1–3	2	1/1	1 (50.0)	2 (100.0)	0 (0.0)
	3–6	4	2/2	1 (25.0)	4 (100.0)	1 (25.0)
	≥6	4	3/1	2 (50.0)	4 (100.0)	0 (0.0)
IgAV	0–1	0	0/0	0 (0.0)	0 (0.0)	0 (0.0)
	1–3	1	0/1	1 (100.0)	1 (100.0)	1 (100.0)
	3–6	6	1/5	4 (66.7)	6 (100.0)	3 (50.0)
	≥6	18	10/8	6 (33.3)	18 (100.0)	2 (11.1)
Other	0–1	4	3/1	2 (50.0)	2 (50.0)	0 (0.0)
	1–3	4	0/4	1 (25.0)	2 (50.0)	0 (0.0)
	3–6	4	1/3	0 (0.0)	4 (100.0)	0 (0.0)
	≥6	3	0/3	1 (33.3)	1 (33.3)	0 (0.0)

Clinical features are not mutually exclusive; individual patients may present with more than one feature. Percentages are calculated within each age subgroup.

**Figure 1:**
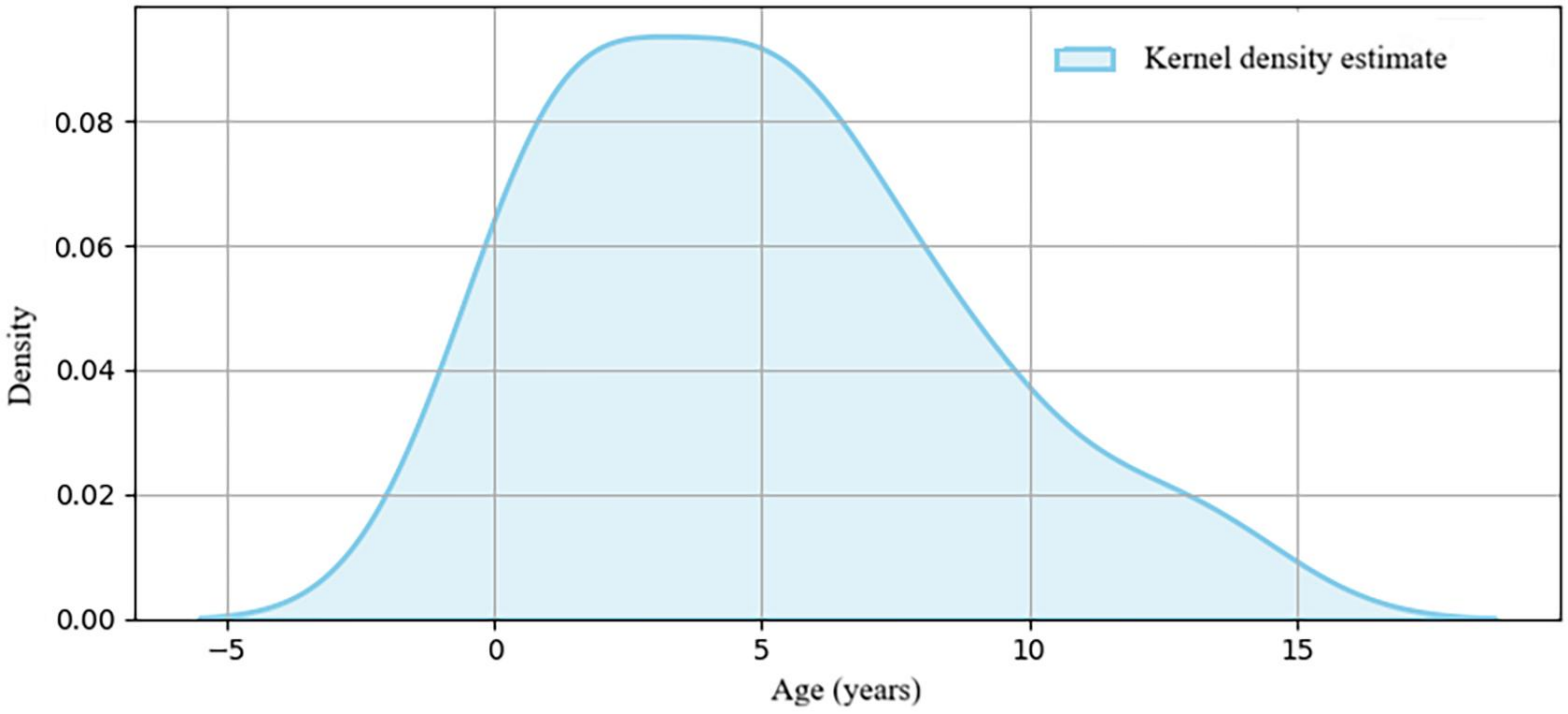
Kernel density estimate (KDE) plot of age distribution in children with intussusception secondary to Meckel's diverticulum.

Meckel's Diverticulum (MD): Primarily affected boys (74.1%), particularly older ones (≥6 years: 88.9% male). These patients were significantly younger than the IgAV group overall (adjusted *p* = 0.011, Table 5). A notable bimodal age distribution was observed, peaking in early childhood (≤2.6 years) and later school age (≥5.0 years) (Figure 1; Table 4), as prespecified, and was statistically supported by Hartigan's dip test (*P* = 0.0174).

**Table 4:** Age distribution characteristics by PLPs.

PLPs	*N*	Age, median (IQR), years	Age range (min-max), years	Description of distribution shape
Intestinal duplication	12	4.0 (2.5–6.2)	0.3–8.0	Approximately symmetric
IgAV	25	7.0 (5.6–8.0)	2.6–12.0	Concentrated distribution (most patients aged 5.6–8.0 years)
MD	27	5.0 (1.6–7.0)	0.2–13.0	Bimodal distribution indicated (Dip test: *P* = 0.017)
Intestinal polyps	13	4.0 (1.0–12.0)	0.1–14.0	Right-skewed distribution; wide age range
Other	15	2.0 (1.0–4.0)	0.6–10.0	Apparent mixed distribution/Heterogeneous distribution

IgA vasculitis (IgAV): Typically presented in older children (≥6 years: 72.0%), who were significantly younger than the intestinal polyp group (adjusted *p* = 0.009, Table 5). Abdominal pain was universally present (100%), while vomiting decreased with age.

**Table 5:** Significant results of pairwise comparisons for clinical characteristics among PLPs groups.

Variable	Comparison group pair	Z-value	Adjusted *P*-value (FDR)	Effect size (*r*)
Age	MD vs. IgAV	−2.95	0.011	0.39
	IgAV vs. intestinal polyps	−3.21	0.009	0.61
Abdominal pain	intestinal duplication vs. Other	1.757	0.032	0.338
	intestinal duplication vs. Intestinal Polyp(s)	2.611	0.003	0.522
	Other vs. IgAV	−2.095	0.003	0.331
	IgAV vs. intestinal polyps	3.077	< 0.001	0.499
	MD vs. intestinal polyps	2.517	0.003	0.403
Vomiting	Other vs. MD	−2.52	0.035	0.389

Intestinal polyps (IP): While predominantly affecting boys (69.2%), this group (*n* = 13) exhibited significantly lower rates of abdominal pain compared to MD, IgAV, and Intestinal Duplication groups (all *p* < 0.003, Table 5). Hematochezia was a frequent finding, particularly in younger children.

Intestinal duplication (ID): Showed a female predominance (*n* = 8/12, 66.7%). Abdominal pain was universal (100%). Abdominal discomfort presented with greater frequency relative to the IP subgroup (*P* = 0.003, Table 5).

### 3.3 Age and gender modifiers within specific etiologies

Within individual PLP subgroups, age and sex emerged as important modifiers of clinical presentation: patients with Meckel's diverticulum showed age-related differences in hematochezia and sex-related differences in abdominal pain, intestinal polyp cases demonstrated age- and sex-associated variations in vomiting, and IgA vasculitis cases exhibited age-related differences in electrolyte disturbance.

Stratified analyses were subsequently performed within each major PLP subgroup to delineate these modifiers in detail (Tables 6, 7). In the Meckel's diverticulum group, the frequency of hematochezia varied significantly with age (*P* = 0.022), and abdominal pain was more common in boys than in girls (95.0% vs. 57.1%, *P* = 0.042). Among patients with intestinal polyps, vomiting differed significantly across age strata (*P* = 0.043) and was markedly more frequent in girls than in boys (100% vs. 22.2%, *P* = 0.021). In contrast, within the IgA vasculitis subgroup, electrolyte disturbances demonstrated significant age-related variation, occurring more frequently in younger children (*P* = 0.030). These intra-group differences underscore the heterogeneity of clinical presentation within specific PLP etiologies and support the need for multifactorial, etiology-oriented diagnostic assessment.

**Table 6:** Significant age-related variations in clinical features within specific PLPs.

PLPs	Clinical feature	*P* - value
MD	Frequency of hematochezia	0.022
intestinal polyps	Frequency of vomiting	0.043
intestinal polyps	Sex distribution across age groups	0.026
IgAV	Occurrence of electrolyte disturbance	0.030
IgAV	Patient age distribution (notably higher prevalence ≥6 years)	0.001

**Table 7:** Significant gender-related differences in clinical features within specific PLPs.

PLPs	Clinical feature	Occurrence rate (Male vs. Female, %)	*P* - value
MD	Abdominal pain	95.0% vs. 57.1%	0.042
intestinal polyps (IP)	Vomiting	22.2% vs. 100%	0.021

### 3.4 Development and performance of preoperative PLP prediction scores

**Figure 2:**
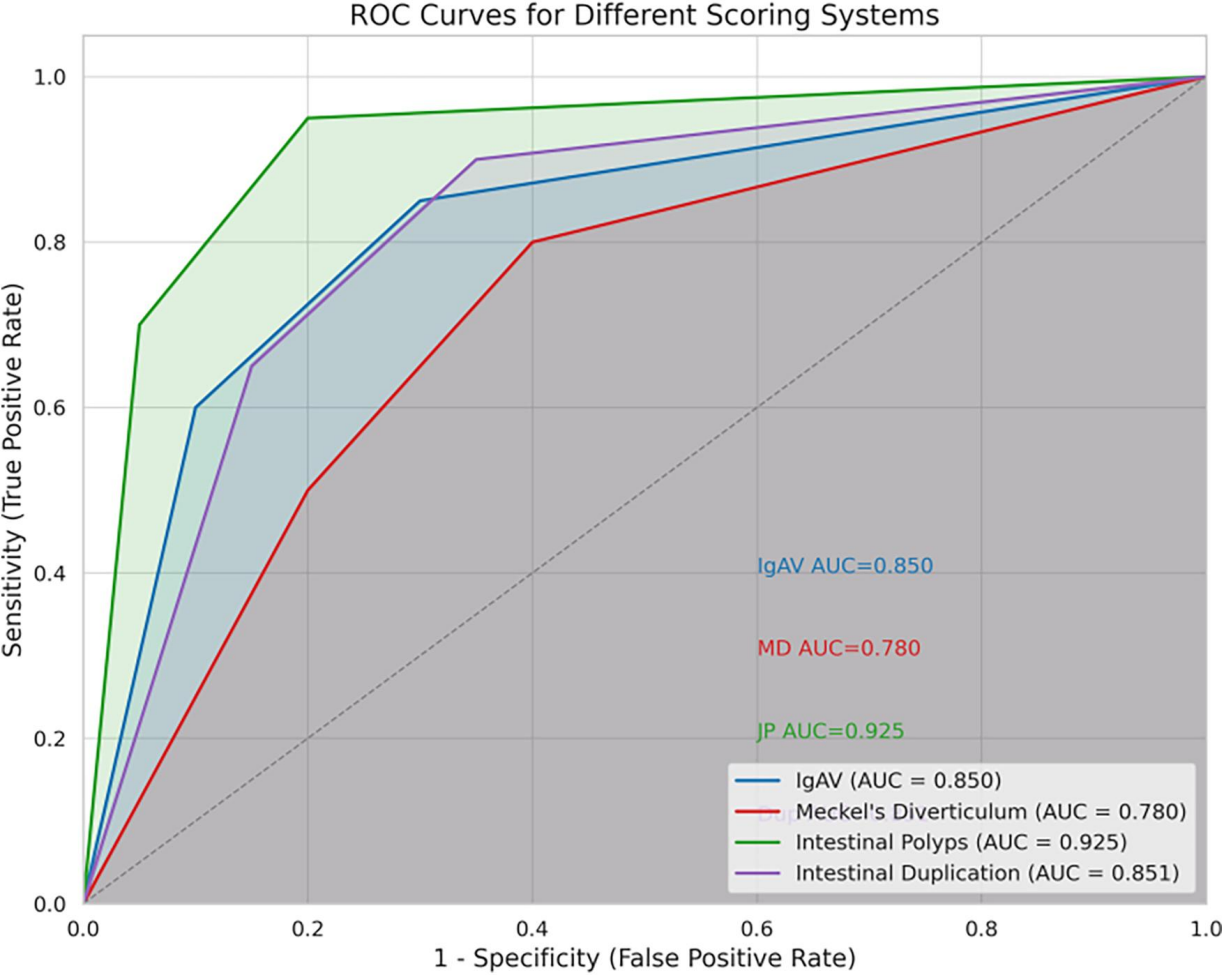
ROC curves for the four scoring systems predicting PLPs in secondary intussusception.

Four simplified etiology-specific scoring systems targeting IgAV, MD, IP, and ID were successfully derived using one-vs.-rest logistic regression, each comprising two to three bedside-available variables and demonstrating moderate to excellent discriminatory performance (AUC range: 0.780–0.925). Based on the methodology described in Section 2.5, the composition and diagnostic performance of each score are summarized below (Table 8; Figure 2).

**Table 8:** Scoring systems developed for preoperative prediction of specific PLPs in pediatric secondary intussusception and their diagnostic performance metrics.

Target etiology score	Variable composition (points assigned)	Total score range	AUC (95% CI)	Cutoff	Sensitivity (%)	Specificity (%)
IgAV Score	Hematochezia (+2), Age ≥6 years (+1), Absence of palpable abdominal mass (+1)	0 to 4	0.850 (0.750–0.932)	3	80.0	75.4
MD Score	Electrolyte disturbance (+1), Vomiting (+1), Male sex (+1)	0 to 3	0.780 (0.682–0.878)	2	84.0	67.7
IP Score	Abdominal pain (–2), Palpable abdominal mass (–1), Electrolyte disturbance (–1)	−4 to 0	0.925 (0.865–0.985)	≥ 0	61.5	90.9
ID Score	Abdominal pain (+1), Male sex (−1), Anemia (−1)	−2 to +1	0.851 (0.742–0.960)	1	75.0	84.6

Scores were derived from etiology-specific one-vs.-rest logistic regression models. For PLP subgroups with limited sample size (intestinal polyps and intestinal duplication), Firth's penalized likelihood estimation was used. Regression coefficients (*β*) were standardized and proportionally scaled, then rounded to the nearest integer to generate point-based scores; negative coefficients were retained as negative point values when applicable. Discriminatory performance was assessed using receiver operating characteristic (ROC) analysis. Optimal cut-off values were determined by the maximum Youden index and rounded to the nearest integer to ensure bedside operability. AUCs shown represent apparent performance in the derivation cohort. Internal validation was conducted using bootstrap resampling with 1,000 iterations. No external validation cohort was available.

Based on the one-vs.-rest (OvR) logistic regression modeling described in Section 2.5, four simplified etiology-specific scoring systems were derived to assist in the preoperative identification of major pathological lead points (PLPs) in pediatric secondary intussusception including IgA vasculitis (IgAV), Meckel's diverticulum (MD), intestinal polyps (IP), and intestinal duplication (ID) (Table 8 and Figure 2). Each score comprised a small set of bedside-available variables selected for clinical interpretability and discriminative performance.

Discrimination was assessed using receiver operating characteristic (ROC) analysis. Using cut-off values derived from the maximum Youden index, the IgAV score—comprising hematochezia (+2), age ≥6 years (+1), and absence of an abdominal mass (+1)—achieved an AUC of 0.850 (95% CI: 0.750–0.932). A cutoff of 3 provided 80% sensitivity and 75.4% specificity.

The MD score included electrolyte imbalance (+1), vomiting (+1), and male sex (+1), yielding an AUC of 0.780 (95% CI: 0.682–0.878). A threshold of 2 demonstrated favorable sensitivity and specificity (84.0% and 67.7%, respectively).

The IP score applied inverse weights for abdominal pain (−2), abdominal mass (−1), and electrolyte imbalance (−1), reaching the highest AUC of 0.925 (95% CI: 0.865–0.985). Using an integer cut-off of 0, the IP score achieved a sensitivity of 61.5% and a specificity of 90.9%. The score demonstrated a high negative predictive value (93.3%), supporting its role in ruling in polyp-related intussusception when positive.

The ID score assigned positive weight to abdominal pain (+1) and inverse weights to male sex (−1) and anemia (−1), achieving an AUC of 0.851 (95% CI: 0.742–0.960). Using a cutoff of 1, the score attained 75% sensitivity and 84.6% specificity.

Overall, these results indicate that compact, etiology-oriented scoring systems can provide meaningful discrimination among major PLP subtypes prior to definitive etiologic confirmation.

## 4 Discussion

### 4.1 Addressing the diagnostic challenge of pediatric secondary intussusception

Secondary intussusception is a high-stakes pediatric emergency in which timely identification of an underlying pathological lead point (PLP) is essential to prevent ischemia, necrosis, and perforation. While ultrasound and contrast enema remain central to confirming intussusception and guiding initial management, etiologic attribution can be difficult in a subset of cases because lead points may be small, intermittently visible, or masked by edema, and diagnostic yield is operator- and context-dependent (11, 12, 14). In this setting, bedside-available clinical patterns mayprovide useful “etiologic priors” after intussusception is confirmed but before definitive etiologic confirmation. Therefore, we applied an “etiology–age–sex–symptom” framework to structure clinical heterogeneity across major PLP subtypes and to translate those differences into simplified, etiology-specific scores intended for preoperative diagnostic prioritization.

### 4.2 Distinct clinical profiles of major PLP etiologies

Across the four common PLPs, we observed that age distribution and symptom phenotype were not interchangeable, supporting the concept that secondary intussusception should not be treated as a single homogeneous entity (10). Importantly, the discriminative features highlighted below align with the variables ultimately retained in our etiology-specific scoring systems, reinforcing their clinical interpretability.

#### 4.2.1 Meckel's diverticulum: age structure and symptom phenotype aligned with the MD score

In our cohort, MD showed a distinct age structure (including a bimodal pattern) and a bedside symptom phenotype that maps onto the MD score, particularly vomiting and electrolyte disturbance, together with male sex. MD is a remnant of the vitelline duct and may contain ectopic mucosa, providing a plausible biologic substrate for bleeding and variable symptom expression across developmental stages, including a characteristic bleeding pattern (e.g., painless fresh hematochezia and/or “currant jelly” stool). Consistent with classic pediatric series describing MD as a frequent and clinically important lead point, our findings support maintaining a high index of suspicion for MD not only in younger children but also in older children when these bedside features suggest higher likelihood (15, 16). From a practical standpoint, when the MD score indicates elevated likelihood, clinicians may consider prioritizing targeted etiologic evaluation—such as repeat/focused ultrasonography and CT when clinically indicated, and, where appropriate (e.g., bleeding pattern), Tc-99 m pertechnetate scintigraphy—rather than relying on nonspecific observation alone (12).

#### 4.2.2 IgA Vasculitis: systemic context and bedside phenotype supporting early etiologic suspicion

IgAV-related intussusception in our cohort clustered in school-aged children (≥6 years) and showed a characteristic systemic bedside phenotype, including purpura rash and arthralgia, often accompanied by abdominal pain. These features are consistent with vasculitic bowel wall edema acting as a functional lead point for intussusception (17, 18). Clinically, once intussusception is confirmed, the presence of such vasculitic manifestations is actionable and may justify early immunologic and renal evaluation, as well as closer clinical monitoring while etiologic clarification is prioritized. Accordingly, our findings are intended to support preoperative etiologic suspicion and diagnostic prioritization rather than prognostic inference (18, 19).

Although IgA vasculitis is classically characterized by systemic manifestations such as purpuric rash and arthralgia, these features were not incorporated into the IgAV-specific score in the present study. Instead, the score was intentionally constructed using a limited set of variables that are consistently available at the bedside after confirmation of intussusception, including hematochezia, age category, and the absence of a palpable abdominal mass. Systemic features such as purpura or joint involvement may provide important contextual clues during clinical assessment and can raise early suspicion for IgAV-related intussusception; however, they are not uniformly documented at the time of surgical triage and were therefore excluded to preserve score parsimony and bedside operability. Accordingly, the IgAV score should be interpreted as a focused adjunct for etiologic prioritization after intussusception has been established, rather than as a comprehensive representation of the full IgAV clinical phenotype.

#### 4.2.3 Intestinal polyps (IP): relatively indolent pain phenotype and implications for endoscopic prioritization

For IP-related intussusception, a key heterogeneity signal in our cohort was a comparatively less prominent pain phenotype, which directly informed the negative weighting of abdominal pain in the IP score. Prior pediatric polyp literature highlights rectal bleeding as a common presenting feature, supporting the clinical plausibility that polyp-driven intussusception may present with bleeding disproportionate to pain severity in some children (9). Accordingly, when the IP score indicates higher likelihood—particularly in children with bleeding and less severe pain—prompt endoscopic evaluation can be prioritized to achieve both diagnosis and potential intervention, while remaining attentive to syndromic features when relevant (20).

#### 4.2.4 Intestinal duplication: imaging-centered confirmation with limited symptom specificity

Intestinal duplication as a PLP is a congenital lesion for which imaging plays a pivotal role in localization and etiologic confirmation (21). In our cohort, bedside symptom patterns offered limited discriminatory value for duplication, reinforcing an imaging-forward approach once intussusception is confirmed. Therefore, when the duplication score indicates higher likelihood, repeat focused ultrasonography (and higher-resolution imaging when clinically indicated) together with early surgical consultation may be appropriate, particularly in atypical or recurrent presentations.

### 4.3 Clinical application: integrating etiology-specific scores into preoperative prioritization

A key contribution of this study lies in translating etiologic heterogeneity into four simplified bedside scores, each composed of two to three routinely available clinical variables and demonstrating moderate to good discrimination (AUC 0.780–0.925, consistent with internal validation results in Section 2.5). Importantly, these scores are intended to support diagnostic prioritization after intussusception is confirmed—helping clinicians determine which etiology to suspect first and which diagnostic pathway to prioritize—rather than to replace imaging, endoscopy, or surgical judgment.

**Figure 3:**
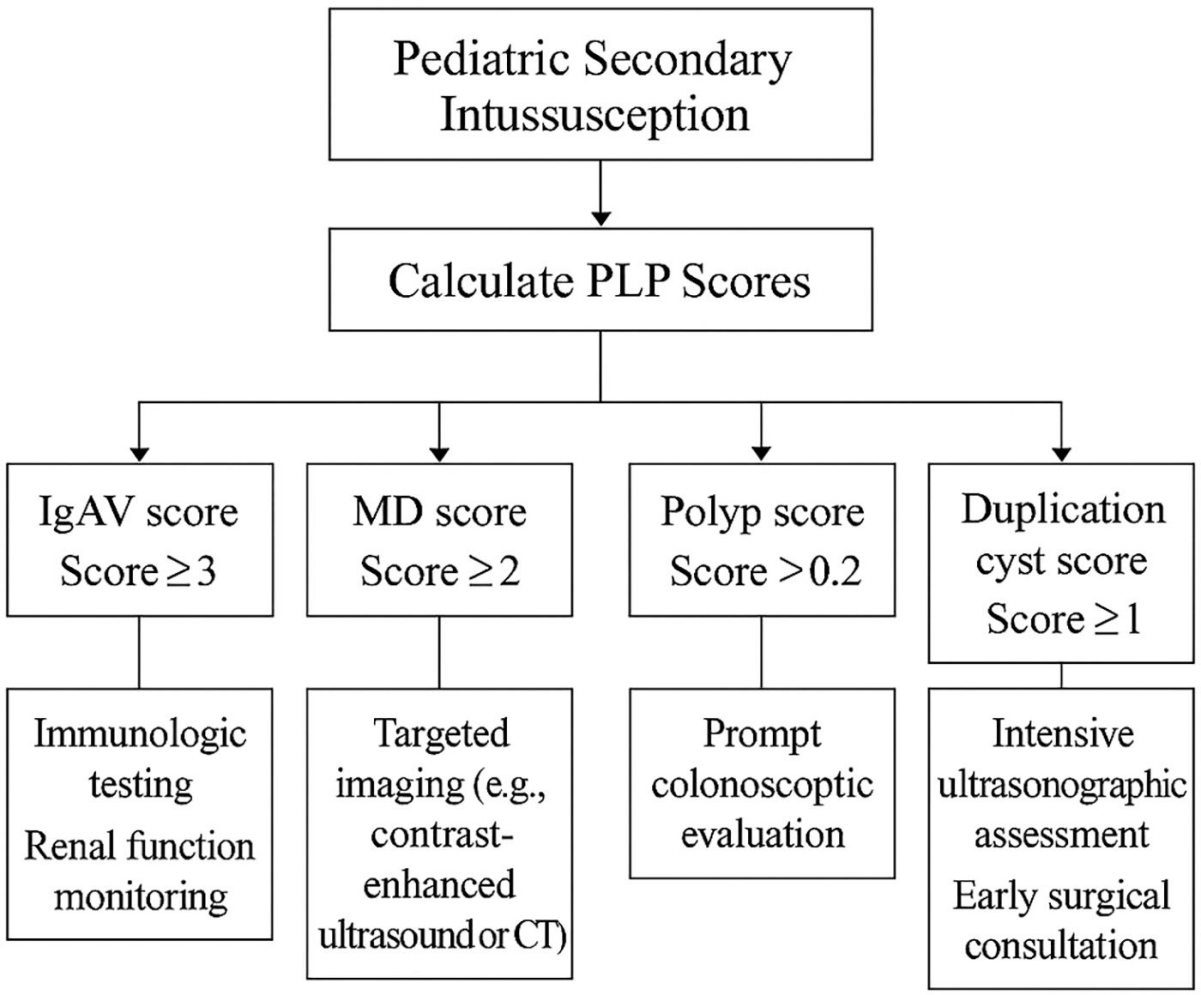
Clinical decision flowchart for major pathological lead points.

In practical terms, an elevated IgAV score may prompt early correlation with systemic vasculitic features and renal monitoring; a higher IP score may justify expedited colonoscopic evaluation; and increased MD or duplication scores may support targeted etiologic imaging strategies, such as repeat focused ultrasonography or CT when clinically indicated, including adjunctive Tc-99 m pertechnetate scintigraphy when bleeding patterns are suggestive (Figure 3; Table 9).

**Table 9:** Proposed cut-off values and suggested diagnostic approaches using etiology-specific scoring systems for PLPs.

PLPs	Proposed cut-off score	Suggested diagnostic approach
IgA Vasculitis (IgAV)	≥3	Correlate score with clinical picture (purpura, arthritis), laboratory work-up (CBC, relevant immune/inflammatory markers), and abdominal ultrasound.
Meckel's Diverticulum (MD)	≥2	Prioritize further imaging: CT scan or Tc-99 m pertechnetate scintigraphy.
intestinal Polyp (IP)	≥0	Proceed promptly with colonoscopy for diagnosis and potential intervention.
intestinal Duplication (ID)	≥1	Perform detailed ultrasound assessment; consider early surgical exploration if suspicion persists or ultrasound is inconclusive.

Given the retrospective, single-center design and the absence of external validation, these tools should be interpreted as triage aids rather than stand-alone decision rules. Prospective, multicenter validation will be necessary before broader clinical implementation.

### 4.4 Limitations

Several limitations warrant consideration. First, the retrospective, single-center design introduces potential selection and information biases and may reflect local diagnostic pathways, limiting generalizability. Second, subgroup stratified by age or sex within specific PLP categories were constrained by sample size and should be regarded as exploratory.

Most importantly, although internal validation (bootstrap resampling) was performed to estimate optimism and assess overfitting risk, the proposed scoring systems have not been externally validated in independent, multicenter cohorts. Future studies should focus on external validation, calibration assessment, and evaluation of clinical impact, as well as on integration with standardized imaging pathways or decision-support tools to determine their real-world utility.

## 5 Conclusion

This study presents a stratified analysis of pediatric secondary intussusception based on underlying pathological lead points (PLPs) and proposes four simplified, etiology-specific scoring systems for IgA vasculitis, Meckel's diverticulum, intestinal polyps (IP), and intestinal duplication. Each score incorporates 2–3 easily assessable clinical variables—such as age, sex, and key symptoms—and demonstrated good to excellent discriminatory performance (AUCs 0.780–0.925) in internal validation. These scoring tools may support early etiologic prioritization, guide imaging strategies, and inform surgical planning in acute pediatric care.

While internally robust, the proposed models require further confirmation in diverse, prospective cohorts to support widespread clinical adoption. Furthermore, future research should explore integrating these tools with imaging modalities, electronic health records, or AI-driven diagnostic platforms to enhance real-time decision support and individualized preoperative assessment.

## Data Availability

The raw data supporting the conclusions of this article are not publicly available due to ethical and privacy considerations, but can be made available by the authors upon reasonable request.
